# Foraging for fast food: the changing diets of wildlife

**DOI:** 10.1093/conphys/cox046

**Published:** 2017-08-09

**Authors:** Essie M. Rodgers

**Affiliations:** 1 Department of Wildlife, Fish and Conservation Biology, University of California, One Shields Avenue, Davis, CA 95616, USA

You are what you eat. So what if all of the food you eat is poor in nutrients, contaminated with pollutants, or blocks your digestive tract? Kim Birnie-Gauvin (2017) and her team reviewed the effects of human activities on wildlife diets, arguing that animals ingest more polluted, unnatural food than ever before.

The base cause of disrupted diets is thought to be pollution—in the atmosphere (e.g., human-driven carbon dioxide emissions), on land, and in water—as well as modifications to natural landscapes for human use. To test this idea, scientists closely monitor the diets of animals. Then, an animal’s gut content or faeces can be examined to identify ingested items and quantify energetic and nutritional value. Researchers typically compare growth, survival or reproductive success of animals feeding on natural food items to animals feeding on unnatural diets, such as crops.

So how does a problem as large as carbon dioxide emissions scale down to influence the daily diets of animals? In plants, elevated carbon dioxide changes the balance between carbon and essential elements (e.g., nitrogen and phosphorus) and diminishes nutritional value. Animals feeding on these plants experience reduced growth, even if they simply eat more to compensate. Polluted environments can have similar effects. For example, in areas where heavy metal pollution is high, birds’ appetites diminish. Fish also experience problems feeding because some pollutants interfere with digestive enzymes.

Rubbish and human food—for example, items from dumps/tips and even fisheries bycatch—are regularly consumed by wildlife. Food subsidies only benefit a handful of species (e.g., the ‘urban exploiters’—rats, gulls and foxes), but most actually suffer. Iguanas and stingrays fed by tourists have poorer body condition compared to animals that are not interacting with tourists. Similarly, seabird chicks that feed on fisheries bycatch will grow more slowly and are less likely to survive than chicks feeding on a normal diet.

But it is not all doom and gloom. Human interventions can also positively influence the diets and health of wildlife. Indeed, making sure nutritious food is available is an important conservation strategy, and if done carefully, can even reverse population declines in ‘at risk species’. One success story saw an increase in reproductive output in the endangered stitchbird when mothers were provided with specially formulated food. The additional food doubled chick survival! Likewise, the endangered kakapo (a large flightless bird) laid more eggs when females were fed nutrient-rich pellets.

The importance of high-quality diets to wildlife health is becoming increasingly recognized and integrated into conservation practices. However, our understanding of the long-term impacts of altered diets in wildlife is lacking. Kim Birnie-Gauvin (2017) and her team stress the need for more research. Additional knowledge will certainly be key to new conservation strategies. And then, we can hope to see the ‘clean-eating craze’ hit the rest of the animal kingdom.

**Figure:**
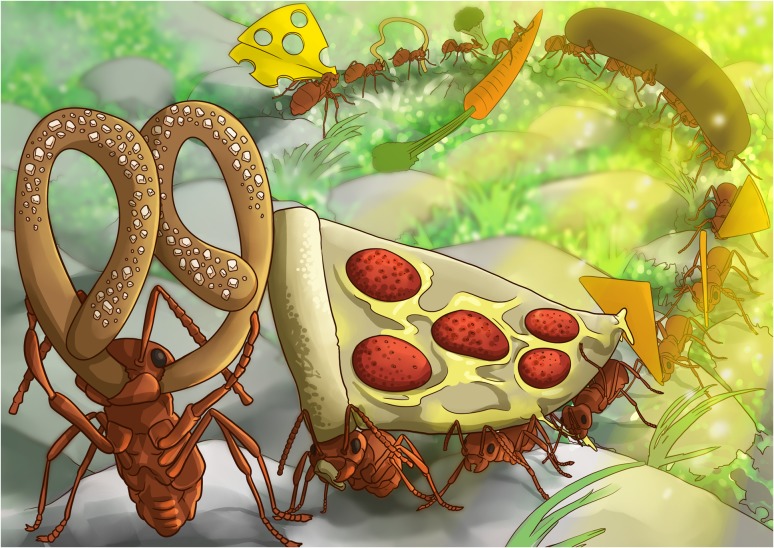


Illustration by Erin Walsh; Email: ewalsh.sci@gmail.com
